# Phylogeography of the antilopine wallaroo (*Macropus antilopinus*) across tropical northern Australia

**DOI:** 10.1002/ece3.2381

**Published:** 2016-10-14

**Authors:** Jessica J. Wadley, Damien A. Fordham, Vicki A. Thomson, Euan G. Ritchie, Jeremy J. Austin

**Affiliations:** ^1^ Australian Centre for Ancient DNAUniversity of AdelaideNorth TerraceAdelaideSouth Australia5005Australia; ^2^ Environment Institute and School of Biological SciencesUniversity of AdelaideNorth TerraceAdelaideSouth Australia5005Australia; ^3^ Centre for Integrative EcologySchool of Life and Environmental SciencesDeakin UniversityBurwoodVictoria3125Australia; ^4^ Sciences DepartmentMuseum VictoriaCarlton GardensMelbourneVictoria3001Australia

**Keywords:** Biogeographic barriers, Carpentarian, divergence, genetics, macropod, marsupial, tropical savannah

## Abstract

The distribution of antilopine wallaroo, *Macropus antilopinus*, is marked by a break in the species’ range between Queensland and the Northern Territory, coinciding with the Carpentarian barrier. Previous work on *M. antilopinus* revealed limited genetic differentiation between the Northern Territory and Queensland *M. antilopinus* populations across this barrier. The study also identified a number of divergent lineages in the Northern Territory, but was unable to elucidate any geographic structure. Here, we re‐examine these results to (1) determine phylogeographic patterns across the range of *M. antilopinus* and (2) infer the biogeographic barriers associated with these patterns. The tropical savannahs of northern Australia: from the Cape York Peninsula in the east, to the Kimberley in the west. We examined phylogeographic patterns in *M. antilopinus* using a larger number of samples and three mtDNA genes: NADH dehydrogenase subunit 2, cytochrome b, and the control region. Two datasets were generated and analyzed: (1) a subset of samples with all three mtDNA regions concatenated together and (2) all samples for just control region sequences that included samples from the previous study. Analysis included generating phylogenetic trees based on Bayesian analysis and intraspecific median‐joining networks. The contemporary spatial structure of *M. antilopinus* mtDNA lineages revealed five shallow clades and a sixth, divergent lineage. The genetic differences that we found between Queensland and Northern Territory *M. antilopinus* samples confirmed the split in the geographic distribution of the species. We also found weak genetic differentiation between Northern Territory samples and those from the Kimberley region of Western Australia, possibly due to the Kimberley Plateau–Arnhem Land barrier. Within the Northern Territory, two clades appear to be parapatric in the west, while another two clades are broadly sympatric across the Northern Territory. MtDNA diversity of *M. antilopinus* revealed an unexpectedly complex evolutionary history involving multiple sympatric and parapatric mtDNA clades across northern Australia. These phylogeographic patterns highlight the importance of investigating genetic variation across distributions of species and integrating this information into biodiversity conservation.

## Introduction

The distribution of a species’ genetic diversity reflects ecological and evolutionary processes operating over different time scales (Hewitt 2000). Quaternary climate change and landscape‐scale biogeographic features shape intraspecific phylogeographic patterns, via shifting habitats that create barriers and isolate populations. These habitat changes influence gene flow, population bottlenecks and genetic drift, which can be detected in species’ current genetic diversity (Taberlet et al. 1998; Hampe and Petit 2005). Investigating present day phylogeographic patterns can provide information on the number and geographic distribution of populations within a species range, thus identifying the relative roles of contemporary verses historical processes that have helped shape modern diversity (Avise 2000).

The tropical monsoon region of northern Australia represents a unique biome comprising a range of habitats and natural landscapes that are relatively intact and unaffected by recent human activities (Potter et al. 2012). The regional climate is characterized by dry winters and wet summers, which has a strong effect on the distribution of plant and animals species (Bowman et al. 2010). The range boundaries of many species are often concordant, suggesting vicariant barriers have played a major role in influencing biogeography in the area (Rollins et al. 2012). To date, phylogeographic analyses [reviewed in (Bowman et al. 2010)] have supported inter‐ and intraspecific vicariance for many species in response to a number of northern Australian biogeographic barriers.

**Figure 1:**
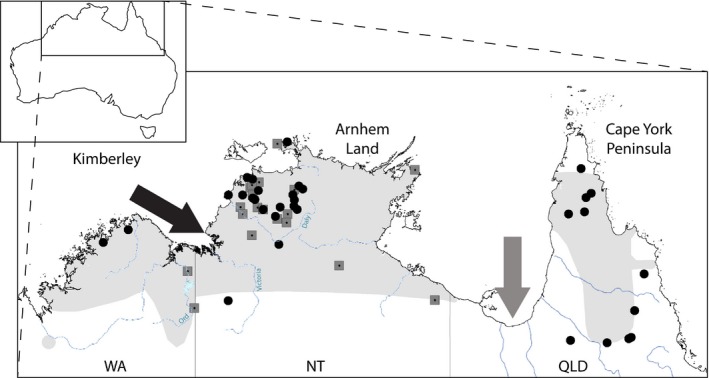
Current distribution (gray shading) of *Macropus antilopinus* across northern Australia showing sample locations (filled circles – samples with three mtDNA gene sequence “3mtgene”, gray squares – samples with control region only “CRonly”). The break in range at the Carpentarian barrier (gray arrow) occurs between Queensland (QLD) and the Northern Territory (NT). The Kimberley Plateau–Arnhem Land barrier between Western Australia (WA) and the NT is shown by a black arrow. Major rivers implicated in the Kimberley Plateau–Arnhem Land barrier are labeled Ord, Victoria, and Daly rivers. For detailed locality data, see Table S1 in Appendix S2.

The antilopine wallaroo (*Macropus antilopinus*) occurs naturally across the tropical savannahs of northern Australia. It exhibits a suite of unique biological traits not found in other macropods, such as a bulbous nose which assists with evaporative cooling in warm, humid conditions (Ritchie 2010). Although *M. antilopinus* is considered by the IUCN to be a “species of least concern” (Woinarski et al. 2008), it is potentially vulnerable to future population declines due to its dependence on tropical grasslands and its vulnerability to climate change (Ritchie and Bolitho 2008). Climate change poses a significant risk to the persistence of *M. antilopinus* as the species’ has a relatively restricted distribution compared to other macropods, high dependence on permanent water sources and seasonal breeding patterns (Ritchie 2007). The distribution of *M. antilopinus* is marked by a break in the species’ range between Queensland (QLD) and the Northern Territory (NT) at the base of the Gulf of Carpentaria (Fig. 1). The Gulf of Carpentaria and the extensive inland area of arid lowland plains of the Gulf country form a biogeographic barrier, known as the “Carpentarian Barrier” or “Carpentarian Gap.” This barrier divides the Cape York Peninsula from the rest of northern Australia (Braby 2008). The barrier is an approximately 150‐km wide, semiarid region extremely poor in vegetation (Lee and Edwards 2008) that is thought to have developed as a result of fluctuations in sea level during the Pleistocene as well as a steep climate and habitat gradient across this area during the last glacial maximum (Cracraft 1986; Lee and Edwards 2008).

Phylogeographic studies on a range of taxa confirm the importance of the Carpentarian barrier in dividing western and eastern populations across northern Australia (Cardinal and Christidis 2000; Jennings and Edwards 2005; Braby 2008; Lee and Edwards 2008; Toon et al. 2010; Kearns et al. 2011; Eldridge et al. 2014). Prior to the most recent marine inundation of the Gulf of Carpentaria [~10,000 years ago (Chivas et al. 2001)], gene flow between populations of *M. antilopinus* in the NT and QLD may have occurred widely across the now inundated Carpentarian plain. Following marine inundation, this gene flow ceased due to unsuitable habitat in this Carpentarian barrier (Ritchie et al. 2008).

Northern Australia also contains a suite of western barriers that may have affected gene flow among *M. antilopinus* populations in the NT and Western Australia (WA). Ford (1978) was the first to propose a “minor” biogeographic barrier splitting Arnhem Land and the Kimberley, referred to as the Bonaparte barrier. More recently additional, geographically proximate barriers have been identified: the “Ord Arid Intrusion” (Bowman et al. 2010), “Bonaparte Gap or Barrier” (Toon et al. 2010), Daly River Drainage Barrier (Potter et al. 2012), and the Victoria River Drainage Barrier (Joseph and Omland 2009). Eldridge et al. (2011) reviewed these barriers and suggested a single term – the Kimberley Plateau–Arnhem Land Barrier – be applied to the whole region, encompassing the four specific barriers. Arnhem Land (NT) and the Kimberley Plateau (WA) are areas of relatively high relief, rainfall, and more mesic woodland, possibly representing more favorable habitat for *M. antilopinus* (Ford 1978; Cracraft 1986) compared to the river catchments separating them (Daly, Victoria and Ord Rivers).

As one of the few large terrestrial mammals restricted to the northern tropics, investigating the phylogeography of *M. antilopinus* is important to gain a broader understanding of historical biogeography in the region and the evolutionary processes shaping diversity in other large terrestrial herbivores in tropical savannahs in southern Asia, central Africa, and northern South America (e.g., Lorenzen et al. 2012). Eldridge et al. (2014) assessed the influence of the Carpentarian Barrier on *M. antilopinus* and its sister species, the common wallaroo (*M. robustus*), which has a continuous distribution across northern Australia. Surprisingly, while there is good evidence for mtDNA and microsatellite divergence for *M. robustus* across the Carpentarian Barrier, there was only limited differentiation between the disjunct NT and QLD *M. antilopinus* populations (Eldridge et al. 2014). This lack of divergence was attributed to a Late Pleistocene (but pre‐LGM) colonization of Cape York by *M. antilopinus* from the NT, with the Carpentarian Barrier subsequently acting as a partial barrier or porous filter. Eldridge et al. (2014) also identified a number of divergent mtDNA lineages in the NT and WA, but were unable to elucidate their geographic structure, due to small sample sizes and limited sampling. Here, we re‐examine these results using additional mtDNA genes and increased sampling on either side of the Carpentarian Barrier and additionally attempt to address the impact of the Kimberley Plateau–Arnhem Land Barrier. Specifically, we use two mitochondrial DNA datasets to (1) determine phylogeographic patterns across northern Australia and (2) infer the biogeographic barriers associated with these patterns.

## Methods

### Sample collection

A total of 89 *M. antilopinus* samples were obtained in the form of DNA extracts (*n* = 19) from previous studies (Ritchie 2007; Eldridge et al. 2014), and from museum collections as cryo‐preserved tissue (*n* = 8) and bone samples (*n* = 62). The DNA extracts are from the same Queensland individuals sampled by Eldridge et al. (2014) matching GenBank numbers: KF974370–KF974382. The additional samples included bone or dry tissue remnants removed from within the nasal cavity of museum skulls. The total dataset encompassed samples from QLD (*n* = 29), the NT (*n* = 44), WA (*n* = 12) and four samples with unknown collection localities. For additional comparison, 32 *M. antilopinus* mtDNA control region haplotypes (KF974382–KF974414) representing 57 additional samples (NT = 53 and WA = 4) were obtained from GenBank (Eldridge et al. 2014).

### DNA extraction, amplification, and sequencing

Three mtDNA fragments were targeted: 1146 bp of cytochrome b (cytb), 1042 bp of NADH dehydrogenase subunit 2 (ND2), and 609 bp of control region (CR). These fragments have been widely used in macropod phylogenetics and phylogeography (Fumagalli et al. 1997; Bulazel et al. 2007; Phillips et al. 2013).

All extractions and PCRs included negative controls to monitor for DNA contamination during the extraction and amplification procedures. DNA was extracted from frozen tissue samples using a “salting out” procedure (Nicholls et al. 2000). All DNA extracts and tissue samples were amplified and sequenced using primers: M441 and M442 for cytb; M1034 and M1035 for ND2; and L15999M and H16498M for CR (Table S1 in Appendix S1). The PCR amplifications were conducted in 25 *μ*L volumes containing 2 *μ*L of DNA, 1× HotMaster PCR Buffer (Eppendorf, Hamburg, Germany), 0.2 mmol/L each dNTP, 0.4 *μ*mol/L of each primer, and 0.5 U HotMaster *Taq* DNA Polymerase (Eppendorf). Thermocycling was conducted using 2 min enzyme activation at 94°C followed by 35 cycles of 94°C for 20 sec, 55°C for 10 sec, and 65°C for 60 sec, with a final extension step of 65°C for 10 min. PCR products were purified using AMPure (Agencourt, Brea, CA) as per manufacturer's instructions and Sanger sequenced with BigDye v3.1 chemistry (Life Technologies, Carlsbad, CA) in 1 of 16 reactions using the original PCR primers and additional internal sequencing primers (Table S1 in Appendix S1). Sequencing reactions were purified using CleanSEQ (Agencourt) as per manufacturer's instructions and run on an in‐house ABI 3130XL Genetic Analyzer (Applied Biosystems, Foster City, CA).

All pre‐PCR work on the historic museum and bone samples were preformed in a dedicated and physically separate clean‐room DNA facility at the Australian Centre for Ancient DNA, University of Adelaide, to control of contamination. No contemporary mammal samples or DNA had ever been present in the pre‐PCR laboratory. The pre‐PCR laboratory protocol included the use of separate dead‐air glove boxes for DNA extraction and PCR setup, regular decontamination of all work areas and equipment with sodium hypochlorite, personal protective equipment including disposable laboratory gown, face mask, hair net, face shield, shoe covers and double‐gloving, and strict one‐way movement of personnel. DNA was extracted from bone samples using a Qiagen DNeasy blood and tissue kit with the following modifications; the amount of ATL, AL, and ethanol added were doubled. Bone extracts underwent amplification and sequencing using species identification primers (Wadley et al. 2013) to determine the success of the extraction and to confirm the species identification as *M. antilopinus* before continuing with the PCRs for the three targeted mtDNA regions. Bone extracts were amplified and sequenced with primers designed to create a number of short overlapping fragments (Table S2 in Appendix S1). PCRs performed with DNA extracts from bones samples were conducted in 25 *μ*L volumes containing 3 *μ*L of DNA, 1× High Fidelity PCR Buffer (Invitrogen), 0.25 mmol/L each dNTP, 2 mmol/L MgSO_4_, 1 mg/mL RSA, 0.4 *μ*mol/L of each primer, and 1 U Platinum^®^ *Taq* High Fidelity (Invitrogen, Carlsbad, CA). Thermocycling was conducted using 2‐min enzyme activation at 94°C followed by 35 cycles of 94°C for 30 sec, 55°C for 30 sec, and 68°C for 30 sec, with a final extension step of 68°C for 10 min. PCR products were then purified using Millipore MultiScreen PCR_384_Filter Plates (Millipore, Billerica, MA) and directly sequenced on an AB 3730*xl* DNA Analyzer (Applied Biosystems) at the Australian Genome Research Facility (AGRF), Adelaide.

Sequence chromatograms were edited and individual sample contigs assembled using Sequencher 4.7 (Gene Codes Corporation, Ann Arbor, MI) and sequences aligned using Geneious Pro 5.6.4 (Biomatters, Auckland, NZ). Two datasets were created; the first contained samples with all three mtDNA regions concatenated (“3mtgene”). The second dataset utilized samples from Eldridge et al. (2014) for which only control region sequence were available, plus all samples from the present study for which control region sequences could be obtained (“CRonly”). This dataset was created to directly compare sequences with those from previous studies and to examine the potential different results obtained using just one mitochondrial gene compared to three. Where needed, one *M. robustus* sample (GenBank: Y10524.1) was included as an out‐group in both datasets.

### Phylogenetic analyses

A phylogenetic tree was constructed for the “3mtgene” dataset to examine the relationships between samples. PARTITIONFINDER version 1.1.0 (Lanfear et al. 2012) was run to determine the appropriate models of sequence evolution for the different mtDNA regions and codons (of the coding genes) used in the phylogenetic analysis. The phylogenetic tree was based on Bayesian analysis and generated using MRBAYES version 3.2 (Ronquist et al. 2012). Four independent runs of Markov chain Monte Carlo analysis were performed with 2,500,000 cycles sampling every 250 cycles. Four chains (1 cold and 3 heated) were used with a temperature of 0.1. The burn‐in was set to 25%, and branch length information was saved on the sampled trees. Summary statistics were produced for all sampled trees and a consensus tree produced containing the branch lengths and interior nodes labeled with support values. The consensus tree was then visualized using FIGTREE version 1.4.0 (http://tree.bio.ed.ac.uk/software/figtree/). NETWORK version 4.6.1.1 (Bandelt et al. 1999) was used with both datasets to create intraspecific median‐joining (MJ) networks to visualize evolutionary relationships between haplotypes within *M. antilopinus*.

### Molecular statistics and demography

Molecular and demographic statistics were calculated for both datasets. ARLEQUIN version 3.5 (Excoffier and Lischer 2010) was used to calculate haplotype diversity (*h*) and nucleotide diversity (*π*) and to determine the number of variable sites, haplotypes, and transitions and transversions. Principle coordinate analysis (PCoA) was conducted in GenAlEx version 6.5 (Peakall and Smouse 2012) based on genetic distances between samples. A hierarchical analysis of molecular variance (AMOVA) of populations was performed in ARLEQUIN to examine the distribution of variation and differential connectivity among populations, regions, and populations within regions. Hypotheses of various population histories and groupings based on results from the phylogenetic analyses were tested and compared.

Historical demographic patterns for the “3mtgene” dataset were inferred using Tajima's D (Tajima 1989) and Fu's Fs statistics (Fu 1997) to test for deviations from neutrality, and by analysis of mismatch distributions with 100 bootstrap replicates in ARLEQUIN. In both statistics a negative result can be interpreted as a population expansion and a positive result as a population bottleneck. The mismatch analysis compares distributions of pairwise nucleotide differences to distributions permuted under the null hypothesis of demographic or spatial expansion. The overall validity of the estimated expansion models was evaluated by the tests of raggedness index (HRI) (Harpending 1994) and the sum of squared differences (SSD) (Schneider and Excoffier 1999). The SSD at a level of *P*‐value ≤0.05 was taken as evidence for departure from sudden population or spatial expansion models. Low HRI values (<0.1) indicate a significant fit between the observed and the expected distributions.

## Results

Initial species identity screening for the 89 *M. antilopinus* frozen tissue and museum samples resulted in eight samples failing to amplify, eight species misidentification (seven *M. robustus* and one *M. fuliginosus*), and 73 samples identified as *M. antilopinus*. All misidentifications and failed amplifications were from museum tissues or bone samples. From the 73 positive *M. antilopinus* samples, CR sequences (597 bp) were subsequently obtained from 57 samples. The ND2 region (945 bp) and cytb region (1104 bp) were successfully sequenced for 52 and 51 samples, respectively. Sequences were trimmed to the shortest length common to all samples after the removal of indels. Overall, 50 samples yielded sequence for all three genes. The concatenated “3mtgene” dataset contained 23 samples from QLD, 25 from the NT and two from WA (Fig. 1 and Table S1 in Appendix S2). One sample (11236A) from the NT was removed from some analyses due to the large number of unique substitutions (62) between it and all other *M. antilopinus* samples (resulting in *n* = 24 samples for NT). Excluding this sample, the remaining 49 *M. antilopinus* sequences contained 74 variable sites that defined 40 distinct mtDNA haplotypes from the 2646‐bp sequence analyzed. The “CRonly” dataset contained 114 samples from QLD (23), NT (84), and WA (7) that represents 67 haplotypes (Tables S2 and S3 in Appendix S2).

### “3mtgene” dataset

**Figure 2:**
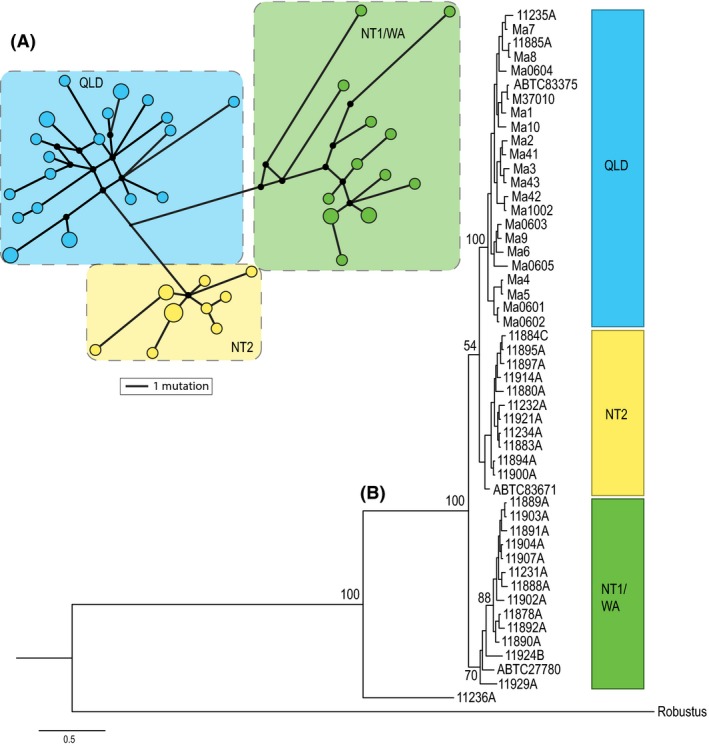
(A) Intraspecific median‐joining (MJ) network for the 40 “3mtgene” dataset haplotypes of *Macropus antilopinus* (representing 49 samples, i.e., excluding divergent 11236A sample). QLD samples are shown in aqua, NT2 in yellow, NT1/WA in green. Circle size is scaled to frequencies of haplotype and branches scaled to the number of mutational steps. (B) MrBayes phylogenetic tree of concatenated mtDNA regions (“3mtgene” dataset) for 50 *Macropus antilopinus* samples (including divergent11236A sample) and one out‐group (*M. robustus*). Posterior probability is shown as branch labels. Green bar represents the NT1 and WA samples, the blue bar represents all the QLD samples, and the yellow bar represents the NT2 samples.

**Figure 3:**
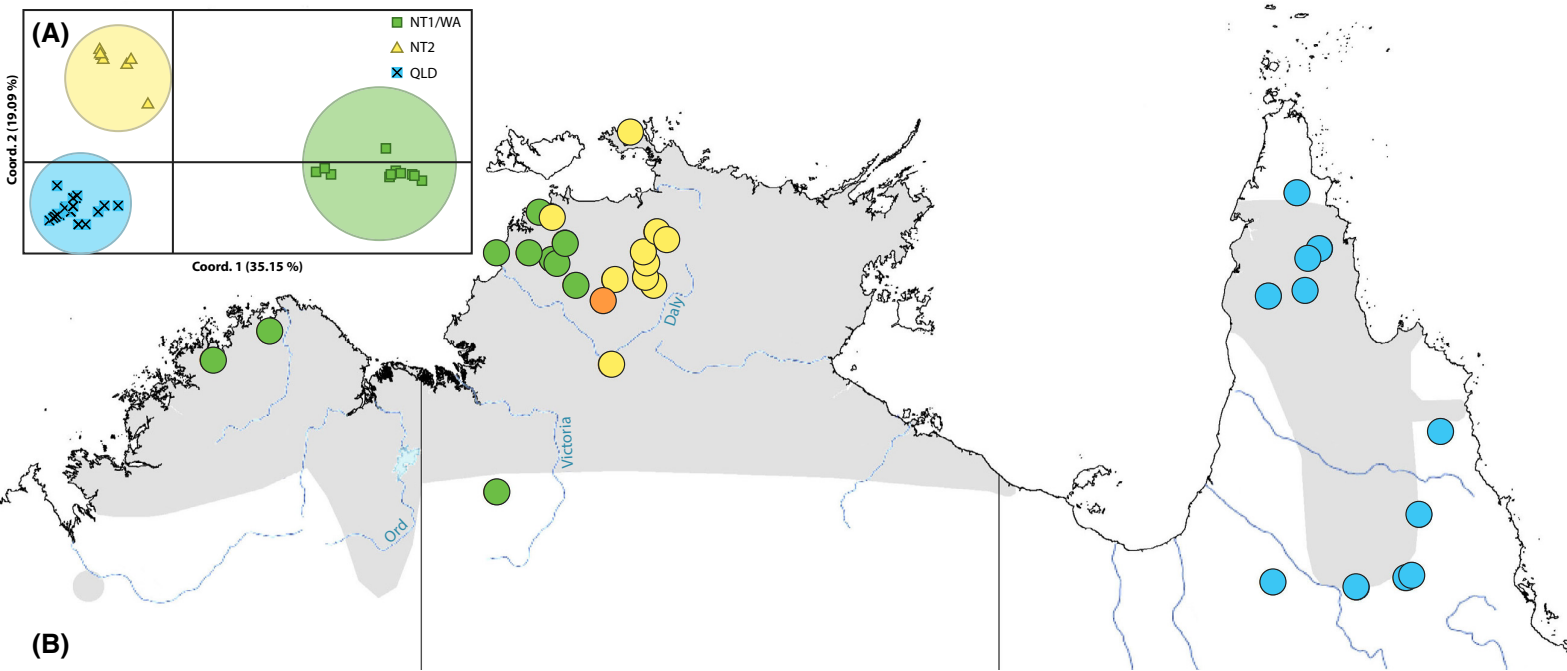
(A) Principal coordinate analysis (PCoA) via covariance matrix of genetic distance for *Macropus antilopinus* samples from the “3mtgene” dataset (49 samples/40 haplotypes, i.e., excluding divergent sample 11236A). QLD samples are shown as aqua crosses, NT2 as yellow triangles, NT1/WA as green squares. (B) Location of *Macropus antilopinus* samples from the “3mtgene” dataset (50 samples/41 haplotypes). Colors indicate the three clades suggested by the phylogenetic analysis. NT1/WA samples are shown in green, NT2 samples are shown in yellow, QLD samples are shown in aqua, and the orange sample is the location of the divergent sample 11236A. Gray shading represents the distribution of *M. antilopinus*. Major rivers implicated in the Kimberley Plateau–Arnhem Land barrier are labeled Ord, Victoria, and Daly rivers.

The phylogenetic tree and haplotype network (Fig. 2) for the concatenated “3mtgene” dataset show samples falling into three clades, a combined Northern Territory and Western Australian group (referred to as NT1/WA), a second Northern Territory group (NT2), and a Queensland group (QLD). Sample 11236A forms a basal lineage to these three main clades. The three clades are also supported by: (1) significant population pairwise *F*_ST_ > 0.5 for all comparisons (NT1/WA vs. NT2 0.69; NT1/WA vs. QLD 0.62; NT2 vs. QLD 0.55); (2) a high degree of differentiation (>19% for coordinate percentage 1 and 2) and clear groupings in the PCoA (Fig. 3); and (3) >32% variance among these groups determined in the AMOVA analysis (Table 1). There was a high level of genetic divergence between the two NT populations (NT1 and NT2) and an absence of any geographic overlap (Fig. 3).

**Table 1:** AMOVA results for plausible populations grouping based on the network and PCoA analyses for *Macropus antilopinus* “3mtgene” dataset (in gray) compared to less likely state‐based and east/west population groupings (in white)

Comparison groupings	ϕ Statistics			Percentage variation		
	FCT (among region)	FSC (pop within region)	*F*_ST_ (among pop)	Among groups	Among populations within groups	Within populations
3 population hypothesis based on network and PCoA						
(QLD) (NT2) (NT1/WA)	0.32851	0.47415*	0.64690**	32.85	31.84	35.31
3 population hypothesis based on states						
(QLD) (NT) (WA)	−0.22333	0.69658**	0.62882**	−22.33	85.21	37.12
2 population hypothesis based on east–west split						
(QLD/NT2) (NT1/WA)	0.32884	0.53495**	0.68787**	32.88	35.90	31.21

*P < 0.05; **P < 0.01.

Tajima's D was significantly negative only for the entire “3mtgene” dataset including 11236A (Table 2). Fu's Fs statistics show significantly negative results for all samples combined (both including and excluding 11236A) as well as in the QLD, NT1, NT2, and combined NT1/WA subgroups, potentially indicating demographic expansion for these groupings (Table 2). Overall, the mismatch distributions for the most plausible population groupings (three clades of QLD, NT2, and combined NT1/WA, excluding 11236A), supported population expansions as expected under sudden (recent) demographic expansion and spatial expansion models (range expansion with high levels of migration between neighboring populations). This is seen in the small SSD and HRI values and a lack of significant evidence against the expansion models in most populations (Table 2) and reflects the unimodal shape of the mismatch distributions (Fig. S1 in Appendix S3). One clade, NT1/WA, produced a significant SSD result against the sudden expansion model, indicating a departure from this demographic model (Table 2 and Fig. S1 in Appendix S3).

**Table 2:** Genetic diversity and historical demographic patterns of *Macropus antilopinus* populations and the complete sample set from across northern Australia using the “3mtgene” dataset The gray boxes are hypotheses supported by the phylogenetic tree, network, and PCoA

Population		Genetic diversity		Neutrality test		MD demographic expansion		MD spatial expansion	
Name	Size (number of haplotypes)	*H*	*π*	Tajima *D*	Fu's Fs	SSD	HRI	SSD	HRI
QLD	23 (19)	0.9842 ±0.0170	0.0024 ±0.0013	−1.2571	−9.8052**	0.0072	0.0209	0.0072	0.0209
WA	2 (2)	1.0000 ±0.5000	0.0060 ±0.0062	0.0000	2.77259	N/A	N/A	N/A	N/A
NT1 (excluding 11236A)	12 (10)	0.9697 ±0.0443	0.0019 ±0.0011	−1.5552	−3.7113*	0.0210	0.0551	0.0207	0.0551
NT2	12 (9)	0.9394 ±0.0577	0.0012 ±0.0008	−1.4384	−3.6439*	0.0286	0.1267	0.0282	0.1267
All (excluding 11236A)	49 (40)	0.9915 ±0.0060	0.0046 ±0.0023	−0.9840	−21.5719*	0.0100	0.0109	0.0142*	0.0109
All (with 11236A)	50 (41)	0.9918 ±0.0058	0.0056 ±0.0028	−1.8433*	−18.7108*	0.0097	0.0101	0.0149	0.0101
3 population hypothesis based on network and PCoA									
QLD	23 (19)	0.9842 ±0.0170	0.0024 ±0.0013	−1.2571	−9.8052**	0.0072	0.0209	0.0072	0.0209
NT2	12 (9)	0.9394 ±0.0577	0.0012 ±0.0008	−1.4384	−3.6439*	0.0286	0.1267	0.0282	0.1267
NT1/WA (excluding 11236A)	14 (12)	0.9780 ±0.0345	0.0027 ±0.0015	−1.3861	−3.9287*	0.1281*	0.0372	0.0272	0.0372

Mismatched distribution (MD), haplotype diversity (H), nucleotide diversity (*π*), raggedness index (HRI), and the sum of squared differences (SSD), *P < 0.05; **P < 0.01.

### “CR only” dataset

**Figure 4:**
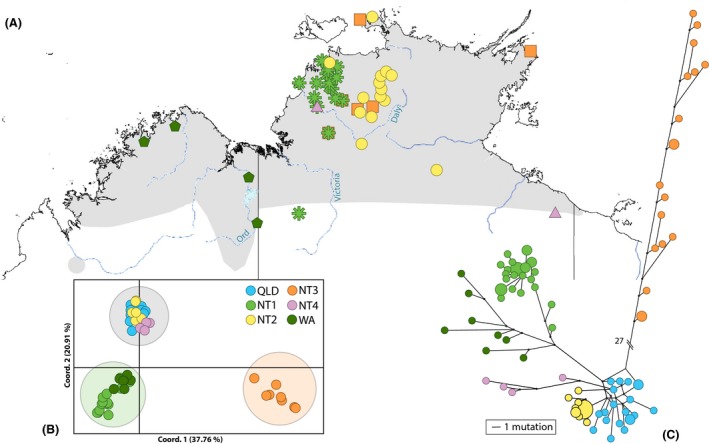
(A) Location of *Macropus antilopinus* samples from the “CRonly” dataset. QLD samples, captive animals, and samples with uncertain collection locality data are not shown (for QLD samples see Figure 3). Samples belonging to NT1/WA are shown in shades of green with dark green pentagons representing WA, and light green asterisks represent NT1 samples. NT2 samples are shown as yellow circles. The orange squares are the highly divergent haplotypes representing NT3. The pink triangles represent the additional control region clade, NT4, from Eldridge et al. (2014). Gray shading represents the distribution of *M. antilopinus* as shown in Figure 1. (B) Principal coordinate analysis (PCoA) via covariance matrix of genetic distance for *Macropus antilopinus* samples from “CRonly” dataset (114 samples/67 haplotypes). QLD samples are shown in blue, NT1 in green, NT2 in yellow, NT3 in orange, NT4 in pink, and WA samples in dark green. Shaded circles indicate larger groupings: NT1/WA green, NT3 orange, QLD/NT2/NT4 gray. (C) Network of “CRonly” dataset comprising 67 haplotypes (114 samples) of *Macropus antilopinus* from this study and Eldridge et al. (2014). QLD samples are blue, NT2 samples are yellow, and NT1/WA samples are green. The green clade has also been split into light and dark to indicate the NT1 samples and WA clade, respectively. The pink and orange clades represent additional NT clades, NT4 samples are in pink, and the highly divergent clade containing 11236A (NT3) is orange.

A reconstruction of the haplotype network using a 583‐bp portion of the control region (largest common fragment length between the generated control region sequences and available GenBank sequences) provides further support for the QLD, NT2, and NT1/WA groupings (Fig. 4). Furthermore, it identified a highly divergent clade containing eight haplotypes/13 samples from the NT (NT3), which included the diverse sample 11236A from the “3mtgene” dataset. An additional NT clade (NT4), containing four haplotypes/six samples, was also found that was not observed in the “3mtgene” dataset. The network also produces a more definitive split within the NT1/WA clade. This resulted in all WA samples (KF974411–KF974414, 11922A, 11924B, 11929A) and one NT sample (KF974398) with uncertain locality data forming a WA clade distinct from the NT1 samples. Significant pairwise *F*_ST_ values were found for the comparisons of all six clades (Table 3). The PCoA analysis also reflects the additional clades but also indicated a separation of clades into three groups (Fig. 4). The three groups are also supported by significant population pairwise *F*_ST_ > 0.5 for all comparisons (NT1/WA vs. QLD/NT2/NT4 0.53; NT1/WA vs. NT3 0.83; QLD/NT2/NT4 vs. NT3 0.78), and >54% variance among these groups determined in the AMOVA analysis (Table S1 in Appendix S3).

**Table 3:** Pairwise population *F*_ST_ values for *Macropus antilopinus “*CRonly” dataset*. F*_ST_ values are below the diagonal. Significant results are shown in bold

	NT1	NT2	NT3	NT4	QLD	WA
NT1	0.000					
NT2	**0.72993**	0.000				
NT3	**0.86330**	**0.86244**	0.000			
NT4	**0.73307**	**0.73965**	**0.78124**	0.000		
QLD	**0.65160**	**0.45648**	**0.82093**	**0.57433**	0.000	
WA	**0.47336**	**0.69650**	**0.76440**	**0.53824**	**0.56174**	0.000

Tajima's D was significantly negative for NT2 and the grouping of NT1/WA for the “CRonly” dataset (Table 4). Fu's Fs statistics show statistically significant negative results for all samples combined as well as in the QLD, NT1, NT2, and combined NT1/WA and QLD/NT2/NT4 subgroups, potentially indicating demographic expansion for these groupings (Table 4). Overall, the mismatch distributions for the most plausible population groupings of six clades and three groups supported population expansions as expected under demographic expansion and spatial expansion models (Table 4). The exception to this were the groups NT2, QLD and the clade grouping of QLD/NT2/NT4 for demographic expansion and also the QLD group for the spatial expansion which produced a significant SSD result against the expansions model indicating a departure from these models (Table 4).

**Table 4:** Genetic diversity and historical demographic patterns of *Macropus antilopinus* populations and the global sample set from across northern Australia for the “CRonly” dataset. The grayed out hypothesis is supported by the phylogenetic network and PCoA

Population		Genetic diversity		Neutrality test		MD demographic expansion		MD spatial expansion	
Name	Size (number of haplotypes)	*H*	*π*	Tajima *D*	Fu's Fs	SSD	HRI	SSD	HRI
NT1	49 (22)	0.9371 ± 0.0203	0.0076 ± 0.0042	−1.1336	−8.4597**	0.0139	0.0268	0.0127	0.0268
NT2	16 (8)	0.7583 ± 0.1104	0.0035 ± 0.0023	−1.6986*	−2.8418*	0.2911**	0.0842	0.0251	0.0842
NT3	13 (8)	0.8974 ± 0.0666	0.01534 ± 0.0085	−0.1229	0.8457	0.0319	0.0651	0.0314	0.0651
NT4	6 (4)	0.8667 ± 0.1291	0.0140 ± 0.0088	0.1973	2.1434	0.1075	0.2044	0.0944	0.2044
QLD	23 (18)	0.9802 ± 0.0176	0.0091 ± 0.0050	−0.8271	−9.5491**	0.0201**	0.0510*	0.0201*	0.0510*
WA	7 (7)	1.0000 ± 0.0764	0.0183 ± 0.0109	−0.8385	−1.4472	0.0194	0.0499	0.0194	0.0499
All	114 (67)	0.9817 ± 0.0051	0.0283 ± 0.0140	−0.4847	−23.8636**	0.0096	0.0039	0.0150	0.0039
3 population hypothesis based on network and PCoA									
QLD/NT2/NT4	45 (30)	0.9636 ± 0.0183	0.0133 ± 0.0070	−0.9165	−13.7020**	0.0181*	0.0189	0.0175	0.0189
WA/NT1	56 (29)	0.9519 ± 0.0163	0.0106 ± 0.0057	−1.5008*	−11.9769**	0.0168	0.0186	0.0164	0.0186
NT3	13 (8)	0.8974 ± 0.0666	0.0154 ± 0.0085	−0.1229	0.8457	0.0319	0.0651	0.0314	0.0651

Mismatched distribution (MD), haplotype diversity (H), nucleotide diversity (*π*), raggedness index (HRI), and the sum of squared differences (SSD), *P < 0.05; **P < 0.01.

To avoid the potential influence of human action, the locality of *M. antilopinus* samples listed as being captive from the Northern Territory Wildlife Park or from Darwin was excluded from the “CRonly” phylogeographic map (Fig. 4). With the exception of the additional clades (NT3 and NT4) and a few geographic outliers, the distributions of the “CRonly” dataset match the results of the “3mtgene” dataset shown in Fig. 3.

## Discussion

Our re‐examination of mtDNA phylogeography in *Macropus antilopinus*, a widespread and vagile macropod, revealed unexpectedly complex genetic patterns that are inconsistent with well‐characterized biogeographic features of northern Australia. Across this monsoonal region, we identified five shallow, but distinct mtDNA clades, with a sixth deeply divergent, basal clade. The broad sympatry of five of these clades across WA and the NT are consistent with predictions that generalist savannah species should show varied and often idiosyncratic histories with higher levels of connectivity, than species restricted to rocky country and drainage systems (Ford and Blair 2005; Bowman et al. 2010). Additional comparative phylogeographic analyses of widely distributed savannah species are needed to further understand the late Quaternary biogeographic history of the monsoon tropics in Australia.

The effect of biogeographic barriers on population differentiation has been investigated in a number of phylogeographic studies across northern Australia (Lee and Edwards 2008; Fujita et al. 2010; Telfer and Eldridge 2010; Toon et al. 2010; Melville et al. 2011; Smith et al. 2011; Potter et al. 2012). Many studies have highlighted the importance of the Carpentarian barrier in shaping species distribution and diversification (Cracraft 1986; Cardinal and Christidis 2000; Jennings and Edwards 2005; Lee and Edwards 2008; Kearns et al. 2011). However, phylogeographic studies of birds have shown contradictory patterns across the Carpentarian barrier: strong differentiation (Lee and Edwards 2008; Toon et al. 2010; Kearns et al. 2011) and the apparent absence of any impact on species differentiation (Kearns et al. 2010; Joseph et al. 2011). We show that the phylogeographic split between QLD and the NT across the Carpentarian Barrier for *M. antilopinus* is shallow.

The separation of QLD lineages coincides with a known split in the species distribution (Fig. 1) and confirms the lack of dispersal across the Carpentarian barrier. This gap in the species’ distribution has been attributed to the formation of the Carpentarian barrier, which contains unsuitable habitat for *M. antilopinus*, and is hypothesized to have prevented subsequent dispersal and gene flow between QLD and NT populations (Ritchie et al. 2008). Prior to the last glacial maximum, the separation of QLD and NT populations is likely to have been initiated by the dramatic drop in sea levels with an associated cooling and increase in aridity across northern Australia (De Deckker et al. 1991). This change in climate caused Lake Carpentaria to spread to its largest extent before intrusion of the sea, possibly affecting dispersal due to unsuitable habitat surrounding the brackish lake. Since the last glacial cycle, Lake Carpentaria merged with rising sea levels to form the Gulf of Carpentaria and the Arafura Sea, severing connections between northern Australia and southern New Guinea. As a consequence, it is predicted that populations of widespread species became fragmented (Braby 2008). Despite the presence of a number of well‐characterized barriers in the Cape York region of north eastern Australia (Lee and Edwards 2008; Toon et al. 2010), there appears to be no phylogeographic structuring of *M. antilopinus* within the Queensland part of its range.

The phylogeographic split between WA and the NT1 across the Kimberley Plateau–Arnhem Land barrier again appears to be very shallow. More extensive sampling from this area and the surrounding Kimberley region would be required to confirm the degree of differentiation and if this region acts as a biogeographic barrier for *M. antilopinus*.

Two clades (NT1 and NT2) appear to be parapatric in western NT, while the remaining two NT clades (NT3 and NT4) are broadly sympatric across the Northern Territory. The separation of the NT1 and NT2 clades does not coincide with known geographic barriers in the area (Joseph and Omland 2009; Potter et al. 2012) but may represent a past vicariant event followed by a secondary re‐colonization by either the NT1 clade from the Kimberley eastward or NT2 clade moving westward, resulting in the two clades coming together (Figs. 3 and 4). Something similar has been hypothesized for rock wallabies in northern Australia where an identical geographic pattern was seen (Telfer and Eldridge 2010). In the arid conditions of glacial maxima, species distributions would have contracted to sites where moisture and food resources remained available. Therefore, the escarpments of Arnhem Land and the Kimberley could have acted as refugia during these times (Telfer and Eldridge 2010). Isolated populations in these refugia would have diverged over time, but then when climates became wetter and food resources more broadly available, *M. antilopinus* would have recolonized previously occupied habitat patches across the landscape. The lack of geographic overlap seen between these clades may be an artifact of a secondary contact zone for two diverged lineages of *M. antilopinus*. Further investigation and additional samples will be required to determine the potential origins and range of the early diverging *M. antilopinus* lineage (11236A in Fig. 2 and NT3 clade in Fig. 4) and the newly identified lineage (NT4 in Fig. 4).

Our results have broader implications for understanding the evolutionary processes that have shaped diversity in other large, widespread macropods in Australia as well as terrestrial herbivores in tropical savannah biomes worldwide. Eldridge et al. (2014) have already highlighted the contrasting biogeographic and phylogenetic patterns across the Carpentarian Barrier between *M. antilopinus* (disjunct distribution, weak genetic differentiation) and *M. robustus* (continuous distribution, strong genetic differentiation). These unexpected patterns hint at species‐specific, idiosyncratic, response to Plio/Pleistocene climate change, that relate to differences in species ecology and/or the location and persistence of “glacial” refugia. Similarly idiosyncratic patterns have been observed in other widespread macropods across Australia, including red kangaroos (*M. rufus*, Clegg et al. 1998), western gray kangaroos (*M. fuliginosus*, Neaves et al. 2012), and eastern gray kangaroos (*M. giganteus*, Zenger et al. 2003). In contrast, codistributed ungulate species in African savannahs show a high level of phylogeographic concordance (Lorenzen et al. 2012) suggesting contraction to and expansion from a common set of savannah refugia. These contrasting patterns indicate that Plio‐Pleistocene climate change in Australia produced a diverse, and sometimes unpredictable, species response, highlighting the need for further studies across a range of vertebrate taxa.

## Conclusion

The contemporary spatial structure of *Macropus antilopinus* gene lineages revealed five clades and a sixth, older lineage. The contemporary genetic differences observed within the four Northern Territory clades, two (NT1 and NT2) being parapatric in western NT, while the remaining two clades (NT3 and NT4) are broadly sympatric, were unexpected. The genetic differences that we found between the Queensland and the Northern Territory *M. antilopinus* samples, confirmed the split in geographic distribution of the species. This split coincides with the Carpentarian barrier representing unsuitable habitat for the species. We also found weak genetic distinctions between Northern Territory samples and those from the Kimberley region of Western Australia, possibly due to the Kimberley Plateau–Arnhem Land barrier. These geographic patterns highlight the importance of investigating genetic variation across species’ distributions. Although presently widespread and not considered threatened, *M. antilopinus* shows clear genetic distinction that will need to be considered in future management and conservation initiatives.

## Conflict of Interest

None declared.

## Supporting information


**Appendix S1.** Primer sequences and reference for primers used in this study.
**Appendix S2.** Museum and location data for samples used in this study.
**Appendix S3.** Additional phylogenetic results including mismatch distributions and AMOVA comparisons.
