# Supplementary material for: Phylogeography of the antilopine wallaroo (Macropus antilopinus) across tropical northern Australia
Source: Ecol Evol. 2016 Oct 14;6(22):8050–61. doi: 10.1002/ece3.2381 (PMC5108257; doi:10.1002/ece3.2381)
Supplement: Supplementary file 1 — Appendix S1. Primer sequences and reference for primers used in this study. Appendix S2. Museum and location data for samples used in this study. Appendix S3. Additional phylogenetic results including mismatch distributions and AMOVA comparisons. [file ECE3-6-8050-s001.docx]

*Journal of Ecology and Evolution*

**SUPPORTING INFORMATION**

**Phylogeography of the antilopine wallaroo (*Macropus antilopinus*) across tropical northern Australia**

Jessica J. Wadley, Damien A. Fordham, Vicki A. Thomson, Euan G Ritchie and Jeremy J. Austin

**Appendix S1** Primer sequences and reference for primers used in this study

Table S1. Primers used to amplify and sequence modern tissue samples and DNA extracts.

| **Primer name** | **Pseudonym** | **Region** | **Sequence 5’ - 3’** | **Reference** |
| --- | --- | --- | --- | --- |
| **External** |  |  |  |  |
| M441 | Ma_cytb_Mr1_F | cytb | CATTTTAGTATGGACTCTAACCATAACC | ([Bulazel *et al.* 2007](#_ENREF_5)) |
| M442 | Ma_cytb_Mr2_R | cytb | AGGGTGTTATACCTTCATTTTTGG | ([Bulazel *et al.* 2007](#_ENREF_5)) |
| M1034 | Ma_ND2_F | ND2 | CCCTTCCCATACTAATGTC | Designed in this study |
| M1035 | Ma_ND2_R | ND2 | GATTTGCGTTCGGATGA | Designed in this study |
| L15999M | Ma_CR_L15999M_F | CR | ACCATCAACTCCCAAAGCTGA | ([Fumagalli *et al.* 1997](#_ENREF_17)) |
| H16498M | Ma_CR_H16498M_R | CR | CCTGAAGTAGCAACCAGTAG | ([Fumagalli *et al.* 1997](#_ENREF_17)) |
| **Internal** |  |  |  |  |
| M462 | Ma_cytb_IntF | cytb | GACAAAGCCACCCTCACACGCT | Designed in this study |
| M463 | Ma_cytb_IntR | cytb | TGTTTCGTGTAGGAATAGGAGGTGGA | Designed in this study |
| M461 | Ma_ND2_IntF3 | ND2 | AACAATGCTCGGAGGCTGAG | Designed in this study |
| M440 | Ma_ND2_IntR | ND2 | AGGCTAGGATTTTTCGTAGGTG | Designed in this study |

Table S2. Primers used to amplify and sequence museum bone samples.

| **Primer name** | **Pseudonym** | **Region** | **Sequence 5’ - 3’** | **Reference** |
| --- | --- | --- | --- | --- |
| A2086 | Ma_cytb_Mr1_F1 | cytb | CATTTTAGTATGGACTCTAACCATAACC | ([Bulazel *et al.* 2007](#_ENREF_5)) |
| A2087 | Ma_Cytb_mus_intR1 | cytb | CGTCTCGGCAGATATGGGCA | Designed in this study |
| A2088 | Ma_Cytb_mus_intF2 | cytb | ATACCTCAGACACCCTAACAG | Designed in this study |
| A2089 | Ma_Cytb_mus_intR2 | cytb | GACAGGAGGTTTGTGATTACGG | Designed in this study |
| A2090 | Ma_Cytb_mus_intF3 | cytb | TGTCCTACCATGAGGACAAAT | Designed in this study |
| A2091 | Ma_Cytb_mus_intR3 | cytb | GATGGTATAATATGGGTGGAATGG | Designed in this study |
| A2092 | Ma_Cytb_mus_intF4 | cytb | TCCGGAATTAACCCCGACTC | Designed in this study |
| A2093 | Ma_Cytb_mus_intR4 | cytb | AAGATGGATGCTAGCAGAGC | Designed in this study |
| A2094 | Ma_Cytb_mus_intF5 | cytb | CCCTAACAAACTAGGAGGAGT | Designed in this study |
| A2095 | Ma_Cytb_mus_intR5 | cytb | GTATGTAGTTTTCAAATAATCCGGC | Designed in this study |
| A2096 | Ma_ND2_mus_intF1 | ND2 | TCAGCCTCTTCCTAGGCACATCCC | Designed in this study |
| A2097 | Ma_ND2_mus_intR1 | ND2 | GGAAGGGGGCCAGGCCTAGTTT | Designed in this study |
| A2098 | Ma_ND2_mus_intF2 | ND2 | ACAGCCTCAGTCCTAATAACCCTAGCA | Designed in this study |
| A2099 | Ma_ND2_mus_intR2 | ND2 | TCCAGCCTATATGGGCGATGGAA | Designed in this study |
| A2100 | Ma_ND2_mus_intF3 | ND2 | TGCTCGGAGGCTGAGGAGGC | Designed in this study |
| A2101 | Ma_ND2_mus_intR3 | ND2 | TGGTATGAAGCCGGTTAGAGGAGGA | Designed in this study |
| A2102 | Ma_ND2_mus_intF4 | ND2 | CCTCCTCACACTTCTATCTCTAGGCGG | Designed in this study |
| A2103 | Ma_ND2_mus_intR4 | ND2 | GGGGTGTTAGTGGGAGTAGGAGGG | Designed in this study |
| A2104 | Ma_CR_L15999M_F1 | CR | ACCATCAACTCCCAAAGCTGA | ([Fumagalli *et al.* 1997](#_ENREF_17)) |
| A2105 | Ma_CR_mus_intR1 | CR | TGAGGTTTAATGTATTTAGTAATGTGTGA | Designed in this study |
| A2106 | Ma_CR_mus_intF2 | CR | GCAATACATAGAATTAATGGTAACTAAGA | Designed in this study |
| A2107 | Ma_CR_mus_intR2 | CR | GTAGTATGTCATTATAGATACGCTAGT | Designed in this study |
| A2108 | Ma_CR_mus_intF3 | CR | ACCAAAACGTGCATAAATGACT | Designed in this study |
| A2109 | Ma_CR_mus_intR3 | CR | ATGGGCCTGCTCTGAAGGAT | Designed in this study |

**Appendix S2** Museum and location data for samples used in this study

Table S1. List of concatenated ‘3mtgene’ dataset *Macropus antilopinus* samples used for phylogeographic analyses showing museum numbers and sample locations.

| **Sample number** | **Museum Accession Number** | **Museum / supplier** | **Sample type** | **State** | **Basic Location** | **Latitude** | **Longitude** |
| --- | --- | --- | --- | --- | --- | --- | --- |
| ABTC27780 | ABTC27780 | ABTC | Tissue | NT | Gregory NP | -16.8045 | 130.166 |
| ABTC83375 | ABTC83375 | ABTC | Tissue | QLD | Mt Surprise | -18.1 | 144.4 |
| ABTC83671 | ABTC83671 | ABTC | Tissue | NT | Katherine | -14.8174 | 131.9504 |
| 11878A | CM5044 | ANWC | Bone | NT | Douglas Station | -13.5833 | 131.4 |
| 11880A | CM1032 | ANWC | Bone | NT | Port Bremer | -11.2 | 132.25 |
| 11883A | CM534 | ANWC | Bone | NT | Kakadu NP | -12.7583 | 132.6567 |
| 11884C | CM552 | ANWC | Bone | NT | Kakadu NP | -13.0667 | 132.45 |
| 11885A | CM757 | ANWC | Bone | QLD | Moreton | -13.1667 | 142.8 |
| 11888A | CM3338 | ANWC | Bone | NT | Darwin | -12.45 | 130.8333 |
| 11889A | CM3402 | ANWC | Bone | NT | Adelaide River | -13.2397 | 131.1065 |
| 11890A | CM5040 | ANWC | Bone | NT | Douglas Station | -13.5833 | 131.4 |
| 11891A | CM5041 | ANWC | Bone | NT | Douglas Station | -13.5833 | 131.4 |
| 11892A | CM5042 | ANWC | Bone | NT | Douglas Station | -13.5833 | 131.4 |
| 11894A | CM5045 | ANWC | Bone | NT | Gimbat Station | -13.5833 | 132.6 |
| 11895A | CM7037 | ANWC | Bone | NT | Kakadu NP | -12.8667 | 132.8 |
| 11897A | CM7120 | ANWC | Bone | NT | Kakadu NP | -12.8667 | 132.8 |
| 11900A | CM7902 | ANWC | Bone | NT | Kakadu NP | -13.4667 | 132.4833 |
| M37010 | M37010 | ANWC | Tissue | QLD | Mt Surprise | -18.1 | 144.4 |
| 11902A | U0018 | MAGNT | Bone | NT | Darwin Area | -12.45 | 130.83 |
| 11903A | U2515 | MAGNT | Bone | NT | Heathers Lagoon | -12.92 | 131.23 |
| 11904A | U2516 | MAGNT | Bone | NT | Stapleton Station | -13.08 | 130.17 |
| 11907B | U2518 | MAGNT | Bone | NT | Stapleton Station | -13.08 | 130.67 |
| 11914A | U5769 | MAGNT | Bone | NT | Palmerston | -12.51 | 131.02 |
| 11231A | M8423 | SAM | Bone | NT | Stapleton Creek | -13.18 | 131.03 |
| 11232A | M276 | SAM | Bone | NT | Mary River | -13.5 | 132 |
| 11234A | M278 | SAM | Bone | NT | Mary River | -13.5 | 132 |
| 11235A | M349 | SAM | Bone | QLD | Coen River | -13.67 | 142.75 |
| 11236A | M8421 | SAM | Bone | NT | Pine Creek | -13.83 | 131.83 |
| 11921A | M17271 | WAM | Bone | NT | Kakadu NP | -13.25 | 132.5 |
| 11924B | M19959 | WAM | Bone | WA | Kalumburu | -14.3 | 126.6333 |
| 11929A | M21259 | WAM | Bone | WA | Mitchell Plateau | -14.75 | 125.75 |
| Ma1 |  | Mark Eldridge | DNA extract | QLD | Rocky Springs Station | -18.1 | 144.4 |
| Ma2 |  | Mark Eldridge | DNA extract | QLD | Kendall River Station | -13.75 | 142.183 |
| Ma3 |  | Mark Eldridge | DNA extract | QLD | Mt Surprise | -18.148 | 144.316 |
| Ma4 |  | Mark Eldridge | DNA extract | QLD | Mt Surprise | -18.148 | 144.316 |
| Ma41 |  | Mark Eldridge | DNA extract | QLD | Georgetown | -18.292 | 143.55 |
| Ma42 |  | Mark Eldridge | DNA extract | QLD | Croydon | -18.205 | 142.249 |
| Ma43 |  | Mark Eldridge | DNA extract | QLD | Croydon | -18.205 | 142.249 |
| Ma5 |  | Mark Eldridge | DNA extract | QLD | Mt Surprise | -18.148 | 144.316 |
| Ma6 |  | Mark Eldridge | DNA extract | QLD | Mt Surprise | -18.148 | 144.316 |
| Ma7 |  | Mark Eldridge | DNA extract | QLD | Merluna Station | -13.0167 | 142.9833 |
| Ma8 |  | Mark Eldridge | DNA extract | QLD | Merluna Station | -13.0167 | 142.9833 |
| Ma9 |  | Mark Eldridge | DNA extract | QLD | Chillagoe | -17.154 | 144.523 |
| Ma10 |  | Mark Eldridge | DNA extract | QLD | Bramwell | -12.1422 | 142.6227 |
| Ma1002 |  | Mark Eldridge | DNA extract | QLD | Mt Surprise | -18.148 | 144.316 |
| Ma0601 |  | Mark Eldridge | DNA extract | QLD | Rocky Springs Station | -18.1 | 144.4 |
| Ma06_02 |  | Mark Eldridge | DNA extract | OLD | Rocky Springs Station | -18.1 | 144.4 |
| Ma06_03 |  | Mark Eldridge | DNA extract | QLD | Lakeland | -15.862 | 144.858 |
| Ma06_04 |  | Mark Eldridge | DNA extract | QLD | Georgetown | -18.292 | 143.55 |
| Ma06_05 |  | Mark Eldridge | DNA extract | QLD | Mt Surprise | -18.148 | 144.316 |

ABTC = Australian Biological Tissue Collection at the South Australian Museum, ANWC = Australian National Wildlife Collection (CSIRO Ecosystem Sciences), MAGNT = Museums and Art Galleries of the Northern Territory, SAM = South Australian Museum, WAM = Western Australian Museum. NT = Northern Territory, QLD = Queensland, WA = Western Australia, NSW = New South Wales, SA = South Australia. NP = national park.

Table S2. Additional *Macropus antilopinus* samples used in ‘CRonly’ dataset for phylogeographic analyses showing museum numbers and sample locations.

| **Sample number** | **Museum Accession Number** | **Museum / supplier** | **Sample type** | **State** | **Basic Location** | **Latitude** | **Longitude** |
| --- | --- | --- | --- | --- | --- | --- | --- |
| 11227A | M8590 | SAM | Bone | NT | Green Ant Creek | -13.52 | 131.20 |
| 11881A | M11311 | ANWC | Bone | ? | Captive Breed CSIRO |  |  |
| 11893A | M05043 | ANWC | Bone | NT | Douglas Station | -13.5833 | 131.4 |
| 11906A | U2517 | MAGNT | Bone | NT | Edith Falls | -14.05 | 132.23 |
| 11912A | U2549 | MAGNT | Bone | NT | Pine Creek | -13.92 | 131.90 |
| 11922A | 17373 | WAM | Bone | WA | Negri River | -17.0666 | 128.9667 |
| 11923B | 17374 | WAM | Bone | NT | South Alligator River | -12.8833 | 132.5000 |

ANWC = Australian National Wildlife Collection (CSIRO Ecosystem Sciences), MAGNT = Museums and Art Galleries of the Northern Territory, SAM = South Australian Museum, WAM = Western Australian Museum. NT = Northern Territory, WA = Western Australia

Table S3. Additional *Macropus antilopinus* samples used in ‘CRonly’ dataset for phylogeographic analyses from GenBank.

| **GenBank number** | **Isolate** | **State** | **Location** | **Latitude** | **Longitude** |
| --- | --- | --- | --- | --- | --- |
| KF974383 | Ma11 | NT | Pungalina | -16.7833 | 137.4667 |
| KF974383 b |  |  | Pungalina |  |  |
| KF974384 | Ma14 | NT | Pungalina | -16.7833 | 137.4667 |
| KF974384 b |  |  | Pungalina |  |  |
| KF974385 | Ma15 | NT | Batchelor | -13.0833 | 131.0333 |
| KF974385 b |  |  | captive |  |  |
| KF974385 c |  |  | captive |  |  |
| KF974386 | Ma17 | NT | Litchfield | -13.5 | 130.6 |
| KF974836 b |  |  | captive |  |  |
| KF974387 | Ma19 | NT | Humpty Doo | -12.6167 | 131.25 |
| KF974387b |  | NT | Darwin River | -12.8167 | 130.95 |
| KF974387 c |  |  | captive |  |  |
| KF974387 d |  |  | captive |  |  |
| KF974387 e |  |  | captive |  |  |
| KF974387 f |  |  | captive |  |  |
| KF974387 g |  |  | captive |  |  |
| KF974388 | Ma20 | NT | Darwin | -13.0833 | 131.0333 |
| KF974388 b |  |  | Batchelor |  |  |
| KF974388 c |  |  | captive |  |  |
| KF974389 | Ma21 | NT | Batchelor | -13.0833 | 131.0333 |
| KF974389b |  | NT | Berry Springs | -12.7167 | 131 |
| KF974389 c |  |  | captive |  |  |
| KF974390 | Ma2_4 | NT | Katherine | -13.75 | 132.2667 |
| KF974391 | Ma2_5 | NT | Daly River | -13.75 | 130.6833 |
| KF974392 | Ma2_8 | NT | Darwin |  |  |
| KF974392 b |  |  | captive |  |  |
| KF974393 | Ma2_10 | NT | Daly River | -13.75 | 130.6833 |
| KF974393b |  | NT | Litchfield | -13.5 | 130.6 |
| KF974393c |  | NT | West Katherine | -14.5 | 131 |
| KF974394 | Ma2_14 | NT | **Daly River **** | -13.75 | 130.6833 |
| KF974394 b |  |  | **Daly River **** |  |  |
| KF974395 | Ma2_15 | NT | **Nutwood/Hodgsons River **** | -15.5667 | 134.0833 |
| KF974396 | Ma2_16 | NT | **Darwin **** |  |  |
| KF974396 b |  |  | **Captive **** |  |  |
| KF974397 | Ma2_18 | NT | Darwin |  |  |
| KF974397 b |  |  | captive |  |  |
| KF974397 c |  |  | captive |  |  |
| KF974398 | Ma2_19 | NT | Darwin |  |  |
| KF974399 | Ma2_21 | NT | captive |  |  |
| KF974400 | Ma2_3 | NT | Coburg | -11.2667 | 131.9 |
| KF974401 | Ma2_24 | NT | captive |  |  |
| KF974402 | Ma2_29 | NT | Gove | -12.1833 | 136.7667 |
| KF974402 b |  |  | Gove |  |  |
| KF974402 c |  |  | Gove |  |  |
| KF974402 d |  |  | Gove |  |  |
| KF974403 | Ma2_31 | NT | Darwin |  |  |
| KF974404 | Ma08_1 | NT | captive |  |  |
| KF974405 | Ma06_6 | NT | West Katherine | -14.5 | 131 |
| KF974406 | Ma08_4 | NT | captive |  |  |
| KF974407 | Ma08_6 | NT | captive |  |  |
| KF974408 | Ma08_9 | NT | captive |  |  |
| KF974409 | Ma08_11 | NT | captive |  |  |
| KF974410 | Ma08_12 | NT | captive |  |  |
| KF974411 | Ma3_4 | WA | Kununurra | -15.7667 | 128.7333 |
| KF974412 | Ma12_1 | WA | Kununurra | -15.7667 | 128.7333 |
| KF974413 | Ma12_2 | WA | Kununurra | -15.7667 | 128.7333 |
| KF974414 | Ma12_3 | WA | Kununurra | -15.7667 | 128.7333 |

NT = Northern Territory. ** samples with conflicting location data between GenBank and Supplementary information from [Eldridge *et al.* (2014](#_ENREF_11))

**Appendix S3** Additional phylogenetic results including mismatch distributions and AMOVA comparisons

a

b

c

Figure S1. Mismatch distributions for *Macropus antilopinus* populations using ‘3mtgene’ database. a = QLD, b = NT2 c = NT1/WA.

Table S1. AMOVA results for plausible populations grouping based on the network and PCoA analyses for *Macropus antilopinus* ‘CR only’ dataset.

| **Comparison groupings** | **φ statistics** | | | **Percentage variation** | | |
| --- | --- | --- | --- | --- | --- | --- |
|  | FCT  (among region) | FSC  (pop within region) | FST  (among pop) | among groups | among populations within groups | within populations |
| **3 population hypothesis based on network and PCoA** | | | | | | |
| (QLD / NT2 / NT4)  (NT1 / WA)  (NT3) | 0.54386 * | 0.49222 ** | 0.76838 ** | 54.39 | 22.45 | 23.16 |
